# Transcription factor SlbHLH70 enhances drought tolerance in tomato

**DOI:** 10.1093/hr/uhag075

**Published:** 2026-03-05

**Authors:** Ang Li, Mayila Yusuyin, Yuping Wei, Chengcheng Shen, Yushun Li, Yafei Li, Xiaoyan Hao, Mairebaike Muhamaitiha, Baike Wang, Juan Wang, Haiyan Lan, Bin Liu, Qinghui Yu

**Affiliations:** College of Life Science and Technology, Xinjiang University, Urumqi 830046, China; Biological Breeding Laboratory, Xinjiang Uygur Autonomous Region Academy of Agricultural Sciences, Urumqi 830091, China; Key Laboratory of Genome Research and Genetic Improvement of Xinjiang Characteristic Fruits and Vegetables, Xinjiang Uygur Autonomous Region Academy of Agricultural Sciences, Urumqi 830091, China; Biological Breeding Laboratory, Xinjiang Uygur Autonomous Region Academy of Agricultural Sciences, Urumqi 830091, China; Biological Breeding Laboratory, Xinjiang Uygur Autonomous Region Academy of Agricultural Sciences, Urumqi 830091, China; Biological Breeding Laboratory, Xinjiang Uygur Autonomous Region Academy of Agricultural Sciences, Urumqi 830091, China; Biological Breeding Laboratory, Xinjiang Uygur Autonomous Region Academy of Agricultural Sciences, Urumqi 830091, China; Biological Breeding Laboratory, Xinjiang Uygur Autonomous Region Academy of Agricultural Sciences, Urumqi 830091, China; Biological Breeding Laboratory, Xinjiang Uygur Autonomous Region Academy of Agricultural Sciences, Urumqi 830091, China; Biological Breeding Laboratory, Xinjiang Uygur Autonomous Region Academy of Agricultural Sciences, Urumqi 830091, China; Key Laboratory of Genome Research and Genetic Improvement of Xinjiang Characteristic Fruits and Vegetables, Xinjiang Uygur Autonomous Region Academy of Agricultural Sciences, Urumqi 830091, China; Key Laboratory of Genome Research and Genetic Improvement of Xinjiang Characteristic Fruits and Vegetables, Xinjiang Uygur Autonomous Region Academy of Agricultural Sciences, Urumqi 830091, China; College of Life Science and Technology, Xinjiang University, Urumqi 830046, China; Biological Breeding Laboratory, Xinjiang Uygur Autonomous Region Academy of Agricultural Sciences, Urumqi 830091, China; Key Laboratory of Genome Research and Genetic Improvement of Xinjiang Characteristic Fruits and Vegetables, Xinjiang Uygur Autonomous Region Academy of Agricultural Sciences, Urumqi 830091, China; Biological Breeding Laboratory, Xinjiang Uygur Autonomous Region Academy of Agricultural Sciences, Urumqi 830091, China; Key Laboratory of Genome Research and Genetic Improvement of Xinjiang Characteristic Fruits and Vegetables, Xinjiang Uygur Autonomous Region Academy of Agricultural Sciences, Urumqi 830091, China

## Abstract

Drought stress profoundly impacts plant productivity worldwide. The roles of basic helix–loop–helix (bHLH) transcription factors are critical in processes of plant growth, development, and stress management. However, roles of specific bHLH genes in tomato, particularly in relation to drought tolerance, remain poorly understood. This research identified *SlbHLH70* as a factor that enhances drought tolerance in tomato. Transgenic lines overexpressing *SlbHLH70* exhibited enhanced drought tolerance and improved post-stress recovery, whereas *SlbHLH70* knockout mutants showed increased sensitivity to drought stress. Further study showed that *SlbHLH70* directly regulated genes associated with abscisic acid (ABA) synthesis, ABA-mediated signal transduction, and root development. Our research identified *SlbHLH70* as an important regulator of drought resistance in tomato, offering a valuable genetic target for enhancing crop resilience to water shortages.

## Introduction

As organisms that do not move, plants are consistently exposed to a variety of abiotic stresses, such as drought, salinity, and extreme temperatures [1]. Among various factors, drought is a primary constraint on global crop productivity, often resulting in larger yield losses compared to other abiotic stresses [2]. To cope with drought conditions, plants have evolved a variety of adaptive mechanisms, such as mediating morphological and structural adjustments, osmotic regulation, and hormonal signaling [3, 4]. Acting as an endogenous messenger, abscisic acid (ABA) plays a role in signaling both biotic and abiotic stresses, particularly as a long-distance indicator of water stress during early drought [5].

The basic helix–loop–helix (bHLH) transcription factor family, ranking as the second most prevalent in plants, features a conserved ~60-amino acid domain comprising two functional segments: an N-terminal basic region that facilitates DNA recognition and a C-terminal HLH motif essential for dimer formation [6–8]. The HLH region forms homologous or heterodimers through hydrophobic action between alpha-helices, allowing the basic region to specifically recognize DNA cis-regulatory elements [9, 10]. Plant bHLH TFs regulate many key biological processes, such as plant development [11], cell fate determination [12], etc. It has been reported that bHLH transcription factors are involved in the transcriptional regulation of plant responses to multiple abiotic stress conditions, including drought, salinity, and cold stress [13, 14].

Regarding root development, bHLH transcription factors also have a profound impact. The bHLH family gene *PtrbHLH66*, derived from clover oranges (*Citrus trifoliata*), when expressed ectopically, boosts seed germination and root development, thereby increasing drought resistance in transgenic *Arabidopsis thaliana*. It enhances plant drought resistance by controlling root development and removing reactive oxygen species [15]. In sweet sorghum, *SbbHLH85* is a key gene in root development. When exposed to salt stress, the overexpression (OE) of *SbbHLH85* notably enhances both the quantity and length of root hairs, as well as Na^+^ absorption, via the ABA and auxin signaling pathways [16].

ABA is pivotal in drought stress response, with complex production and signaling pathways involving many essential genes. During the rate-limiting step of ABA biosynthesis, the *NCED* (9-cis-epoxycarotenoid dioxygenase) gene is responsible for encoding essential enzymes [17, 18]. Under drought stress, all genes in the CYP707A family are up-regulated; interestingly, the mRNA levels of these genes increase significantly after rehydration [19]. For *cyp707a2* mutants, the seeds remain excessively dormant, with ABA concentrations reaching up to six times those found in wild-type (WT) plants [19]. Therefore, the CYP707A family genes play a role in regulating the decrease of ABA levels in plants.

Tomato (*Solanum lycopersicum*) has become one of the most extensively cultivated and economically significant vegetable crops globally due to its rising presence in international trade in recent years [20, 21]. However, the growth, developmental processes, and final yield of tomato are significantly restricted by various abiotic stresses such as drought [22, 23]. This study employed genetic techniques to demonstrate the crucial role of the *SlbHLH70* gene in enhancing drought tolerance in tomato plants. The study found that *SlbHLH70* gene OE significantly improves tomato plants' drought tolerance, whereas its knockout markedly reduces their drought resilience. Subsequent studies demonstrated that SlbHLH70 regulates multiple key physiological processes associated with drought stress. The relevant downstream genes are involved in ABA synthesis, ABA signaling pathway regulation, and tomato root development.

## Results

### Expression of *SlbHLH70* is influenced by drought stress factors

**Figure 1:**
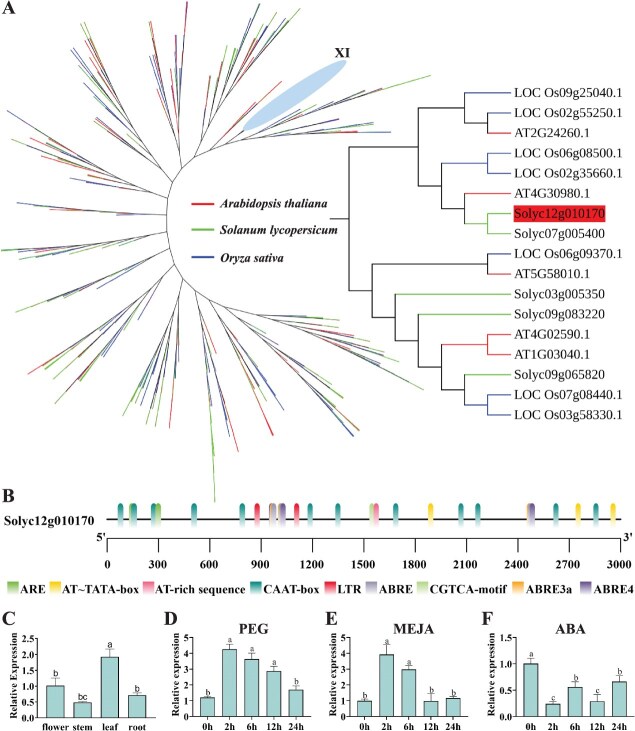
The expression of *SlbHLH70* related to drought tolerance. (A) Neighbor-joining (NJ) phylogenetic tree of bHLH family proteins from *Arabidopsis thaliana* (AtbHLH), *Oryza sativa* (OsbHLH), and *Solanum lycopersicum* (SlbHLH). *SlbHLH70* is clustered in Group XI (highlighted). (B) Predicted cis-elements in promoter regions of *SlbHLH70* genes. Different cis-elements are indicated with distinct markers. (C) Tissue-specific expression of *SlbHLH70* in 6-week-old tomato plants. Relative expression levels in leaves, stems, roots, and flowers were normalized to *SlACTIN1*. (D-F) The time-course expression of *SlbHLH70*. (D) 90 μM MeJA (E) 10 μM ABA (F) Data represent mean ± SE (*n* = 3). Different lowercase letters denote significant differences (*P* < 0.05, one-way ANOVA with Tukey’s post hoc test).

bHLH transcription factors have been widely studied and characterized in various plant species. In *A. thaliana*, the bHLH family has been classified into 17 major taxa (I-XVII) and 31 subfamilies [9, 24]. To elucidate the evolutionary relationship of *SlbHLH70*, we carried out multiple sequence alignment and phylogenetic analysis using bHLH proteins from three model species: tomato (159 genes), Arabidopsis (173 genes), and rice (178 genes). The resulting phylogenetic tree grouped these genes into 11 distinct clades, with *SlbHLH70* assigned to the XI group (Fig. 1A). Although the functions of tomato bHLH genes in this group remain largely unknown, orthologues in *Arabidopsis* are believed to be involved in root hair development and lateral root initiation [10, 25]. *Ljrhl1-1* and *Ljrhl1-2,* two group XI bHLH genes from Lotus japonicus, have been identified as contributors to root hair development [26]. Based on these findings, we hypothesized that *SlbHLH70* may perform similar functions in tomato. Promoter sequence analysis identified multiple cis-acting regulatory elements implicated in development, stress responses, and hormone signaling pathways (Fig. 1B and Supplementary Table S1). Based on expression data, *SlbHLH70* expression was predominantly localized to root tissues, with comparatively lower levels detected during fruit development (Supplementary Fig. S1).

To further elucidate the functional relevance of *SlbHLH70*, we examined its spatiotemporal expression profile. This analysis revealed pronounced transcript accumulation in leaves of 6-week-old tomato plants, contrasting with substantially lower expression levels in stem tissues at the same developmental stage (Fig. 1C). We then assessed the transcriptional response of *SlbHLH70* to treatments with 100 mM polyethylene glycol (PEG), 100 μM ABA, and 100 μM methyl jasmonate (MeJA).

The expression of *SlbHLH70* was rapidly induced by PEG and MeJA within 2 hours post-treatment, approximately increasing by 4-fold, and its levels gradually decreased over time, reaching nearly normal levels by 24 hours (Fig. 1D and E). In contrast, the response of SlbHLH70 to ABA treatment was opposite and more complex; its expression decreased rapidly to about 25% of the initial level within 2 hours, partially recovered thereafter, but showed a second decrease between 6 and 12 hours, and remained below baseline at 24 hours (Fig. 1F). These results suggest that *SlbHLH70 is* responsive to both PEG and MeJA, and may participate in the drought stress response via an ABA-dependent signaling pathway.

### *SlbHLH70* is essential for drought tolerance in tomato

**Figure 2:**
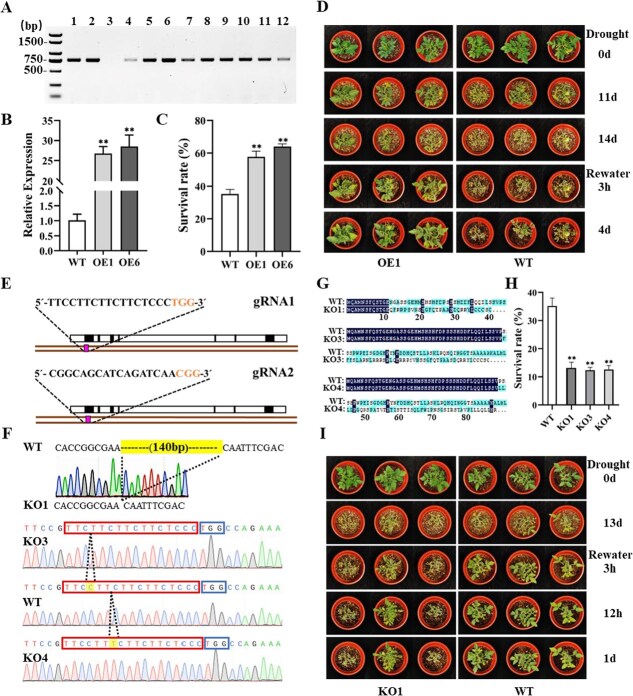
*SlbHLH70* is essential for drought tolerance. (A) PCR to detect specific exogenous sequences. (B) qRT-PCR analysis of SlbHLH70 expression in WT and OE plants. **P < 0.01 (Student's *t*-test). (C) Survival rates of WT and *SlbHLH70* OE plants after 4 days of rehydration following 14-day drought stress (*n* = 30 plants per line). (D) Phenotypic comparison of WT and OE plants under drought stress (0, 11, 14 days) and recovery (3 hours, 4 days). (E) Illustration of the *SlbHLH70* gene structure and the two gRNAs in exon 1. (F) Sanger sequencing results of *SlbHLH70* KO lines (KO1: 140 bp deletion; KO3: 1 bp deletion; KO4: 1 bp insertion). Yellow boxes indicate edited regions. (G) Amino acid alignment of the *SlbHLH70* target region in WT, KO1, KO3 and KO4. (H) Survival rates of WT and knockout plants following a 1-day rehydration period (*n* = 30). (I) Phenotypic comparison of WT and knockout plants under drought and recovery conditions.

To delineate the physiological role of *SlbHLH70* in drought adaptation, we developed transgenic *Solanum lycopersicum* ‘Micro-Tom’ (MT) lines constitutively overexpressing this transcription factor. PCR amplification of partial sequences from the 35S promoter and *SlbHLH70* coding region confirmed the presence of transgenes in the regenerated plants. (Fig. 2A). The detection results of real-time fluorescence quantitative PCR (qRT-PCR) showed that, compared with the WT plants, the transcript abundances of *SlbHLH70* in the two OE lines (OE1 and OE2) were approximately 26-fold and 27-fold higher, respectively (Fig. 2B).

**Figure 3:**
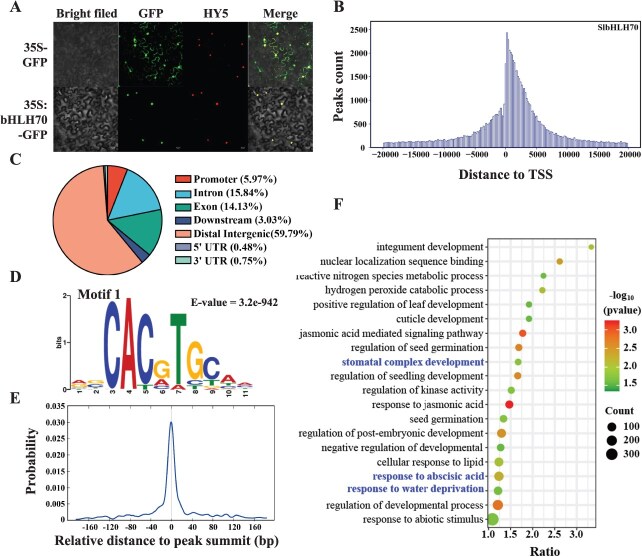
Identification of the genome-wide potential direct targets of *SlbHLH70*. (A) Subcellular localization of SlbHLH70-GFP fusion protein in *Nicotiana benthamiana* epidermal cells. HY5-RFP marks nuclei. (B) Distribution of SlbHLH70 binding peaks relative to transcription start sites (TSS). Peaks are enriched within 2 kb upstream of TSS. (C) Top enriched motif (CACGTG, E-value = 3.2e-942) identified by MEME analysis. (D) Enriched motif within the SlbHLH70 binding peaks. The most significantly enriched core sequence is represented by Motif 1, consisting of 6 nucleotides (5′-CACGTG-3′). The E-value was calculated by MEME. (E) Centrimo analysis of Motif 1, showing the position of motifs relative to the DAP-seq peak centers. The graph shows the average density of motif positions for all motif-containing regions from −160 bp to +160 bp relative to the DAP-seq peak summit. (F) The top 20 enriched GO terms of SlbHLH70’s direct targets.

Based on the above results, we evaluated the drought tolerance of *SlbHLH70* OE lines and WT plants that had grown in soil for 30 days. Natural drought treatment was used to treat the two genotypes. At the beginning of the treatment, the two genotypes of plants had the same growth trend (Fig. 2D). However, when the natural drought stress treatment reached the 11th day, phenotypic differences began to appear between the two genotypes of plants. The leaves of WT plants began to show a wilted phenotype, while the overexpressing plants were relatively much healthier. When the natural drought stress continued until the 14th day, both genotypes of plants showed wilted symptoms. We observed the degree of leaf wilting and the degree of petiole drooping. By comparison, the overexpressing plants were less affected by drought than the WT plants (Fig. 2D).

All the plants were subsequently subjected to rehydration treatment to assess their recovery status. Three hours after rehydration, the OE lines exhibited a pronounced recovery phenotype (such as unfolding of curled leaves), whereas the WT plants showed no significant recovery phenotype. Following a 4-day rehydration period, approximately 60% of the wilted *SlbHLH70* OE plants survived, compared to less than 40% survival in the wilted MT plants (Fig. 2C and D). These findings indicate that OE of *SlbHLH70* enhances drought tolerance and improves recovery capacity after rehydration in tomato. Four days after rewatering, we counted the survival rate of plants under experimental treatments. The survival rates of the two OE lines were both around 60%, which were significantly higher than those of WT plants (less than 40%) (Fig. 2C and 2D). The above results indicate that OE of the *SlbHLH70* gene may enhance the drought tolerance of tomato plants and also improve their recovery ability after rewatering.

To learn more about the role of *SlbHLH70* in resilience to drought, we used CRISPR/Cas9 to create gene KO lines. Genome-edited plants carrying the Cas9 gene were examined for mutations at the target sites of two guide RNAs (gRNAs) that were specially tailored to target the first exon of SlbHLH70 (Fig. 2E). Three independent homozygous mutants were selected, KO1 (140 bp deletion), KO3 (1 bp deletion), and KO4 (1 bp insertion) (Fig. 2F). The protein products generated from the edited gene sequences were predicted using the Open Reading Frame Finder tool in NCBI (https://www.ncbi.nlm.nih.gov/orffinder), and all three mutations resulted in premature termination of translation. Subsequent BLAST analysis of the truncated proteins showed no significant similarity to known proteins (Fig. 2G).

We then evaluated drought tolerance in T1 plants from these mutant lines alongside WT plants grown in soil for 30 days. At the beginning of the drought treatment, KO lines showed growth phenotypes similar to WT plants (Fig. 2I). However, compared to the WT plants, the KO lines had more pronounced drought-induced damage by Day 13 of drought stress, including leaves with drooping petioles. For recovery, all of the plants were irrigated once more. The WT plants had a noticeable recovery phenotype (such as the unfolding of curled leaves) 3 hours after rehydration, whereas the KO plants had not yet displayed any discernible recovery phenotype (Fig. 2I). After an additional day of recovery, approximately 35% of the withered WT plants survived, while less than 15% of the withered KO plants survived (Fig. 2H).

These findings suggest that *SlbHLH70* positively contributes to drought tolerance in tomato. OE of *SlbHLH70* enhances drought resistance and post-stress recovery, while reduced function results in heightened susceptibility to drought and hindered recuperation after rehydration.

### Genome-wide identification of potential direct targets for SlbHLH70

SlbHLH70, a bHLH transcription factor, likely influences abiotic stress responses by modulating downstream target gene expression. According to the nuclear marker of HY5, the GFP-tagged SlbHLH70 protein, which was temporarily expressed in *Nicotiana benthamiana* leaves, was localized in the nuclei of epidermal cells, aligning with its role in transcriptional regulation (Fig. 3A).

DNA affinity purification sequencing (DAP-seq) was employed to identify the genome-wide binding sites of SlbHLH70 and to determine its direct transcriptional targets [27]. In this assay, sheared genomic DNA from 6-week-old WT plants was affinity-purified using an *in vitro*-synthesized SlbHLH70 protein, and deep sequencing was then performed [28].

Analysis of SlbHLH70 DNA-binding sites revealed a strong enrichment near transcription start sites (TSSs), with 5.97% of the peaks located within the 2-kb upstream of the TSS (Fig. 3B and C). This pattern supports the typical role of TFs in regulating gene expression through promoter binding. Analysis with the MEME suite identified a highly enriched binding motif (Motif 1) with the core sequence ‘CACGTG’ within the SlbHLH70 binding regions (E-value = 3.2e^−942^; Fig. 3D), alongside nine additional motifs with lower enrichment (Supplementary Table S2). A more detailed investigation into the distribution revealed that Motif 1 was mostly found within the top of the SlbHLH70 binding peak, suggesting it represents a high-confidence binding motif (Fig. 3E).

Interestingly, Motif 1 closely resembles canonical bHLH binding motifs identified in previous studies, including the regulator of cotton abiotic stress tolerance, *GHbHLH12* [29], and the jasmonic acid (JA) responsive repressor in *A. thaliana*, *ATbHLH017* [30]. Gene ontology (GO) enrichment analysis of SlbHLH70-bound genes revealed significant functional categories associated with drought stress adaptation (Fig. 3F). Enriched GO terms, such as response to water deprivation (GO:0009414) and response to ABA (GO:0009737), indicate that SlbHLH70 directly affects genes related to osmotic stress signaling and ABA-mediated pathways.

**Figure 4:**
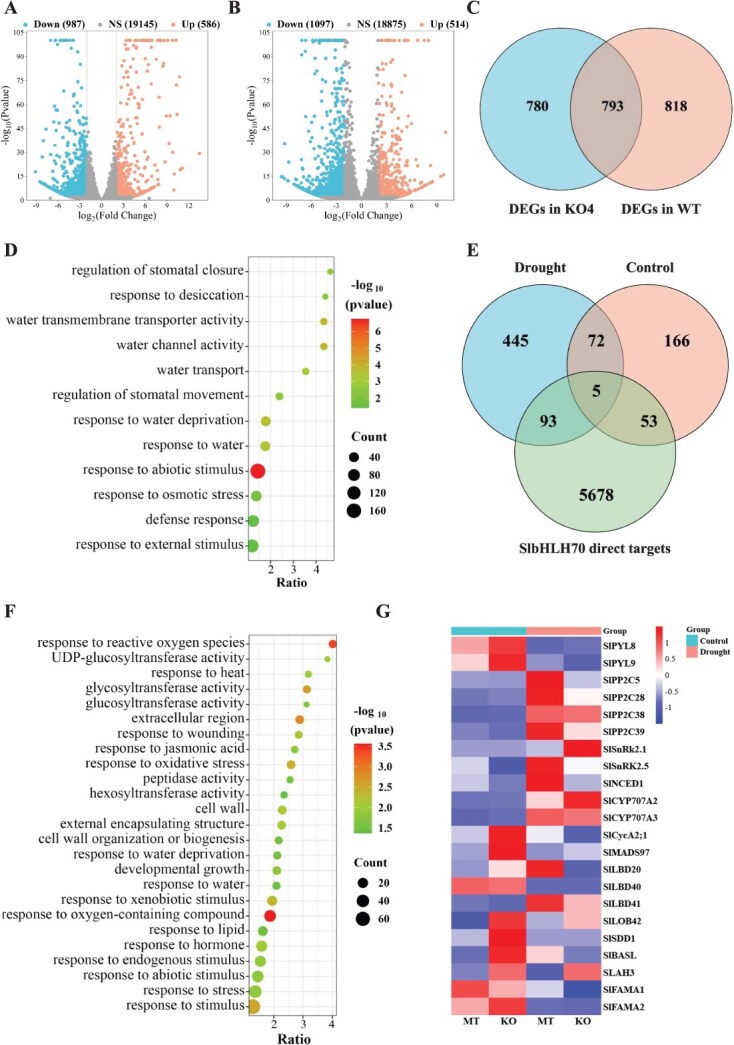
Identification of drought-responsive genes directly regulated by *SlbHLH70.* (A-B) Volcano plot of DEGs in *SlbHLH70* KO (KO4) (A) and WT plants (B) under drought stress and normal watering conditions (|log_2_ FC| ≥ 2, padj ≤0.01). Dots represent upregulated/downregulated genes. (C) Venn diagram comparing DEGs in KO4 and WT under drought stress. (D) GO enrichment analysis of SlbHLH70-dependent DEGs. (E) Integrative analysis of DAP-seq peaks and RNA-seq data. 151 genes with SlbHLH70-bound promoters and drought-responsive expression are highlighted. Venn diagram. ‘Drought’ denotes DEGs between *SlbHLH70* KO and WT plants under drought stress; ‘control’, those under normal watering; SlbHLH70 direct targets, potential target genes from DAP-seq. (F) GO enrichment analysis of the 151 *SlbHLH70* direct target genes identified through integrative DAP-seq and RNA-seq analysis. (G) Heatmap of expression patterns for selected drought-responsive SlbHLH70 target genes under drought stress. Clusters highlight genes with significant upregulation or downregulation in SlbHLH70 KO (KO4) compared to WT plants. Data are normalized to log₂ fold change (|log₂FC| ≥2, padj ≤0.01).

Notably, terms such as regulation of seed germination (GO:0010029) and hydrogen peroxide catabolic process (GO:0042744) were also enriched, suggesting SlbHLH70 may coordinate both developmental and oxidative stress responses under drought. Collectively, these findings support a central role for SlbHLH70 in orchestrating a multifaceted drought response through direct transcriptional regulation of stress-responsive genes.

### SlbHLH70 directly regulates drought-responsive genes

A comparative transcriptome study was conducted using RNA-seq for the leaves of the WT and *SlbHLH70* KO lines under control and drought stress conditions in order to discover drought-responsive genes that are directly controlled by SlbHLH70. Under drought stress, 1573 and 1611 differentially expressed genes (DEGs) were identified in the KO and WT plants, respectively (padj ≤0.01, |log_2_FC| ≥ 2) (Fig. 4A and B and Supplementary Table S3). Comparative analysis of drought-induced DEGs showed that 780 and 818 genes were present only in SlbHLH70 mutant and WT plants, respectively (Fig. 4C). Additionally, we detected 296 DEGs between KO and WT plants under control conditions, and 615 DEGs under drought stress (Supplementary Table S3 and Fig. S2).

To investigate the regulatory role of SlbHLH70, GO enrichment analysis was conducted using DEGs from both the KO vs. WT comparison and the unique drought-responsive genes (Fig. 4C and D). Enriched terms, such as ‘response to water deprivation’ (GO 0009414), indicate that SlbHLH70 is involved in drought stress response.

To identify the direct downstream targets of SlbHLH70, we integrated DEGs obtained from DAP-seq promoter-targeted genes and RNA-seq analysis. SlbHLH70 binds to the promoters of 151 genes, which were transcriptionally regulated under drought stress conditions (Fig. 4E). GO enrichment of these targets showed significant overrepresentation of drought-related terms, and functional classification revealed genes encoding transporters, transcription factors, signaling components, metabolic enzymes, and others (Fig. 4F).

Heatmap analysis highlighted distinct expression patterns: genes, such as *SlPYL8*, *SlSnRK2.1*, and *SlCycA2;1,* were significantly upregulated in SlbHLH70-OE plants but downregulated in KO lines under drought, while stress-negative regulators (e.g. *SlCYP707A2*) showed the opposite trend (Fig. 4G). These results demonstrate that SlbHLH70 functions as a key transcriptional regulator that activates drought-responsive genes while repressing stress attenuation pathways, thereby fine-tuning drought adaptation in tomato.

### SlbHLH70 modulates drought stress responses by regulating genes involved in ABA synthesis and signaling in tomato

**Figure 5:**
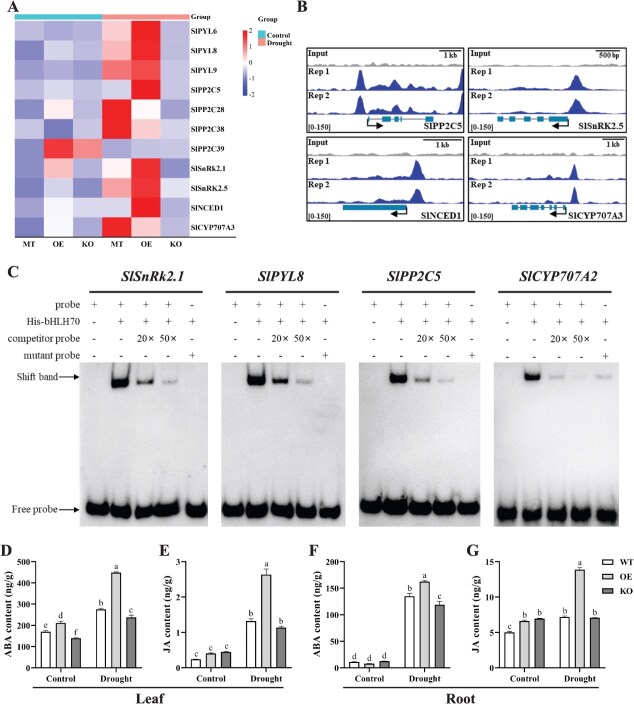
*SlbHLH70* influences gene expression associated with ABA synthesis and signaling in drought-stressed tomatoes. (A) Expression profiles of a set of ABA biosynthesis and signal transduction-related genes affected by *bHLH70* expression in tomato plants. (B) Localization of SlbHLH70 enrichment peaks in the promoter regions of genes involved in ABA synthesis and signal transduction. Arrows mark the translation start sites and their directions. ‘Input’ represents the sequencing results of genomic DNA fragments used as negative control input. ‘Rep’ represents the sequencing results of genomic DNA fragments used as input for the target region of the target gene. (C) EMSA assay showed that SlbHLH70 directly binds to the promoter probes of *SlSnRk2.1*, *SlPYL8*, *SlPP2C5*, and *CYP707A2*. The recombinant HIS-SlbHLH70 protein was incubated with biotin-labeled DNA fragments. (D-G) Endogenous ABA (A and C) and JA (B and D) levels in leaves and roots of WT, OE, and KO plants under drought stress. **P < 0.01 (Tukey's test).

We thoroughly examined the regulatory effects of SlbHLH70 on genes involved in ABA production and signaling in order to clarify the molecular mechanisms underlying drought tolerance. First, we characterized the expression patterns of key drought-responsive genes in *SlbHLH70* OE, KO, and WT plants under control and drought conditions. (Fig. 5A). *SlbHLH70* exerted pronounced control over ABA biosynthesis and signaling components. Under drought stress, OE plants showed enhanced expression of the ABA receptor genes *SlPYL6*, *SlPYL8*, and *SlPYL9*, whereas KO plants showed reduced and blocked expression of each of these receptor genes. The ABA biosynthesis gene *SlNCED1*, which encodes a rate-limiting enzyme in ABA synthesis, showed a strong upward trend in OE plants under drought conditions, while in KO plants, its transcriptional abundance was lower than in WT plants (Fig. 5A). A number of genes were chosen for IGV visualization detection in order to gain a better understanding of the transcriptional control mediated by SlbHLH70. The promoter regions of these genes had significantly enriched SlbHLH70 binding peaks (Fig. 5B).

To verify the direct regulatory relationship of SlbHLH70 on downstream genes, based on the core binding motif (CACGTG) identified by DAP-seq, key candidate genes related to the ABA synthesis and signaling pathway were selected, and the in vitro binding ability of *SlbHLH70* to the target gene promoters was verified by electrophoretic mobility shift assay (EMSA). The core genes of the ABA signaling pathway, *SlSnRK2.1*, *SlPYL8*, *SlPP2C5*, and the ABA catabolic gene *SlCYP707A2* were selected. Promoter probes containing the core G-box motif (CACGTG) were synthesized and incubated in vitro with the recombinant HIS-SlbHLH70 protein. The results showed that *SlbHLH70* could specifically bind to the promoter probes of *SlSnRK2.1*, *SlPYL8*, *SlPP2C5*, and *SlCYP707A2*, forming obvious DNA-protein complex bands (Fig. 5C). The results of the competition experiment showed that the unlabeled WT probe (cold probe) could competitively bind to SlbHLH70 in a concentration-dependent manner (20×, 50×), gradually reducing the intensity of the complex bands. In contrast, the probe with a mutated core motif (mutant probe) could not effectively compete, and there was no significant change in the complex bands. These findings confirm that SlbHLH70 directly binds to these genes by recognizing the G-box motif in the promoter region and is involved in the transcriptional regulation of ABA synthesis and signal transduction.

*SlbHLH70*’s expression level changed when ABA and MeJA were administered (Fig. 1E and F), and various genes involved in plant hormone production, catabolism, and downstream signaling pathways were identified as possible target genes of SlbHLH70 (Fig. 4F). We used liquid chromatography–tandem mass spectrometry (LC–MS/MS) to measure endogenous ABA and JA levels in the leaves and roots of *SlbHLH70* transgenic and WT plants in order to evaluate the physiological significance of these transcriptional alterations. In our detection results, the ABA concentration in leaves was higher than that in roots, whereas the converse was true for JA (Fig. 5D and E). Higher ABA content is typically associated with enhanced drought resistance [31]. Under drought stress, the ABA level in *SlbHLH70-*overexpressing plants was significantly higher than that of WT plants, while the ABA level in knockout (KO) plants was significantly lower than that of WT plants (Fig. 5D and F). Drought stress also led to an increase in JA levels. Compared to WT plants, the JA level in *SlbHLH70*-overexpressing plants was significantly higher under drought stress. However, there were no significant differences in JA levels in leaves and roots between WT and KO plants (Fig. 5E and G).

### SlbHLH70 responds to drought stress by regulating the expression of root development-related genes in tomato

**Figure 6:**
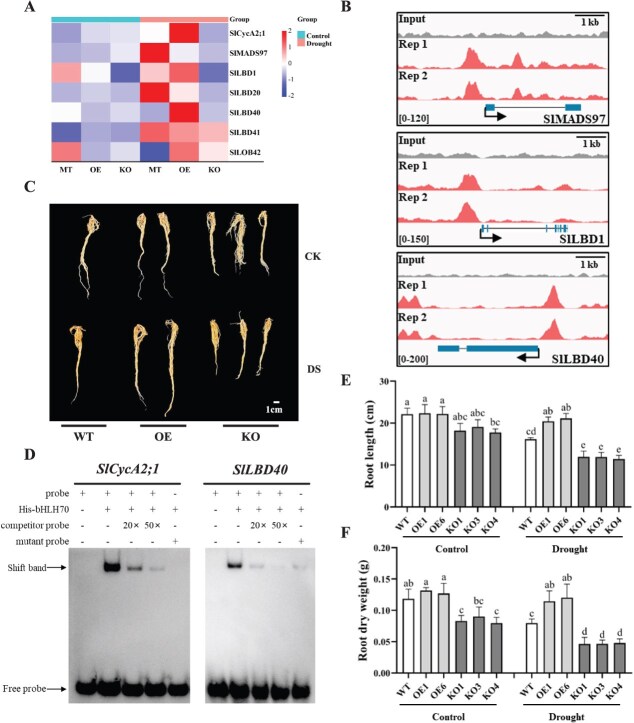
*SlbHLH70* affects the expression of genes related to root development in tomatoes under drought stress*.* (A) The expression profiles of a set of genes related to root development that are affected by the expression of *bHLH70* in tomato plants. (B) Localization of SlbHLH70 enrichment peaks in the promoter regions of root development-related genes. Arrows indicate the translation start sites and their directions. ‘Input’ denotes the sequencing results of genomic DNA fragments used as the negative control. ‘Rep’ represents the sequencing results of genomic DNA fragments corresponding to the target regions of the genes of interest. (C) Root morphologies of WT and transgenic plants subjected to 9-day drought stress. Scale bar: 1 cm. (D) EMSA revealed that SlbHLH70 directly binds to the promoter probes of SlCycA2;1 and SlLBD40. The recombinant HIS-SlbHLH70 protein was incubated with biotin-labeled DNA fragments. (E and F) Effect of natural drought on MT and SlbHLH70 transgenic tomato seedling root systems (n = 10). (D) Root length of SlbHLH70 OE, KO and MT tomato seedlings under natural drought for 9 d and normal watering. (E) Root dry weight of *SlbHLH70* OE, KO, and MT tomato seedlings under natural drought for 9 days and normal watering.

Plasticity of the root system, a crucial characteristic for water uptake during drought, is directly impacted by *SlbHLH70*. In tomatoes, meristem activity and lateral root initiation are linked to *SlMADS97* and *SlLBD40*, respectively [32–34]. Drought caused these genes' expression levels to rise in OE lines while falling in KO lines (Fig. 6A). The binding peaks in the promoters of *SlMADS97*, *SlLBD1*, and *SlLBD40* were visualized by IGV (Fig. 6B). To verify the direct regulatory relationship of SlbHLH70 on downstream target genes, based on the core binding motif (CACGTG) by DAP-seq and, key candidate genes related to root development were selected, and the in vitro binding ability of SlbHLH70 to the promoters of target genes was verified by EMSA. For the key root development genes *SlCycA2;1* and *SlLBD40*, promoter probes containing the G-box motif were also designed for EMSA verification. The results showed that after incubating the recombinant HIS-SlbHLH70 protein with the promoter probes of *SlCycA2;1* and *SlLBD40*, clear migration lag bands appeared, indicating that there was a direct in vitro binding between them (Fig. 6D). The results of specificity verification showed that with the increase in the concentration of unlabeled WT probes, the DNA–protein complexes gradually decreased, while the mutant probes had no competitive activity. This further proved that SlbHLH70 regulates the transcription of SlCycA2;1 and SlLBD40 by specifically recognizing the G-box motif, and participates in the regulation of tomato root development. During drought and recovery, plants overexpressing SlbHLH70 developed more vigorous roots than WT plants, while the roots of SlbHLH70 KO plants showed the opposite trend (Fig. 6C). The differences in root weight and root length demonstrated this (Fig. 6E and F).

In conclusion, SlbHLH70 enhances drought tolerance in tomato primarily by regulating the transcription of genes associated with ABA biosynthesis, ABA signal transduction, and root development.

## Discussion

With climate change, drought stress has led to a significant decrease in crop productivity, and overcoming drought stress has now become one of the key issues in current agricultural development [35]. As one of the important gene families in eukaryotes, the bHLH transcription factor family is widely involved in plant growth and development, metabolic regulation and responses to biotic and abiotic stress [34, 36, 37]. The role that bHLH proteins have in tomato drought response has also been demonstrated [38, 39]. The tomato bHLH transcription factor gene SlbHLH70, which reacts to ABA, MeJA, and drought, was discovered in this work (Fig. 1E–F). Under drought stress, SlbHLH70 OE plants had higher ABA content than WT plants, and root biomass was also significantly increased, while the opposite was observed in SlbHLH70 KO plants (Figs 5 and 6). Because SlbHLH70 regulates ABA signaling and root growth, overexpressing it in tomatoes increased their resistance to drought, while knocking it down decreased their resistance.

Consisting of two alpha helices with hydrophobic residues, the HLH region plays a vital role in the dimerization process. This process alters the expression of target genes involved in diverse signaling pathways. Meanwhile, the basic region contributes to DNA binding with the E-box (usually CANNTG) or G-box (CACGTG) motifs within their target genes [40, 41]. In this study, we analyzed and concluded using the DAP-seq method that the most significantly enriched motif sequence in the binding peak of SlbHLH70 is ‘CACGTG’, which is the same as the previously reported sequence of bHLH binding element G-BOX (Fig. 3D) [42]. *A. thaliana* AtbHLH112 can regulate downstream gene expression in reaction to abiotic stress via binding to G/E-boxes to improve abiotic stress tolerance [43]. In addition, bHLH transcription factors, with AtMYC2 (bHLH6) and AtMYC3 (bHLH5) forming cis-tetramers and homodimers respectively, are crucial in the JA signaling pathway, and the differences in their oligomerization states lead to different regulatory capabilities, while the formation of tetramers and multiple G-box sites are also of great significance for gene regulation [44].

By binding to the G-box and E-box of the *PeTIP1–1* and *PePHT1–1* promoter regions, the bamboo bHLH gene *PeRHL4* controls gene expression, aids in *Phyllostachys pubescens*' water and phosphorus transport, and increases the plants' resistance to adversity [45]. In this study, SlbHLH70 also exhibited an important function in tomato drought stress response, suggesting that bHLH genes may be conserved in plant drought resistance mechanisms. Thus, via binding to G-boxes, *SlbHLH70* is probably going to control the expression of pertinent target genes, which will impact how tomatoes react to drought stress. This mechanism echoes the regulation of other known bHLH proteins, further supporting the reliability of the results.

A key factor in how plants react to drought stress is the ABA signaling system [46]. SlbHLH70 significantly impacted the expression of genes linked to the ABA signaling pathway in this investigation. In SlbHLH70-overexpressing plants, the expression of ABA receptors PYL6, PYL8, and PYL9 was increased, while in KO plants, the expression was decreased (Fig. 5A). The expression patterns of SlPP2C family genes exhibited complex changes in both overexpressed and knockout plants, potentially linked to PP2C's negative regulatory function in the ABA signaling pathway. Furthermore, the SlNCED1 gene, responsible for encoding a crucial enzyme in ABA biosynthesis, showed increased expression in overexpressed plants and decreased expression in knockout plants, thereby directly influencing ABA synthesis.

Under drought stress, SlbHLH70 responds to MeJA (Fig. 1E), and its OE increases the accumulation of JA (Fig. 5E and G). This may interact with ABA signaling to enhance drought tolerance. JA can promote the biosynthesis of ABA and act synergistically with it through common transcription factors (such as MYC2, NACs), thereby enhancing osmotic adjustment [47]. In turn, ABA may fine-tune the JA-mediated signaling cascade, forming a coordinated regulatory network in SlbHLH70-mediated drought adaptation [48]. In rice, bHLH transcription factors (such as OsbHLH148) interact with JAZ proteins to mediate synergistic or additive effects between JA and ABA signals [34, 49]. This synergy may co-regulate downstream stress-responsive genes through shared transcription factors (such as bHLH, MYC), thereby enhancing drought tolerance, suggesting that SlbHLH70-mediated drought adaptation may involve the synergistic action of JA and ABA signaling networks [50].

In addition to acting through the ABA signaling pathway, SlbHLH70 may also regulate the response of tomatoes to drought stress through other pathways. *SlbHLH70*-overexpressing plants exhibited more robust root systems than WT plants throughout drought and recovery, with notable variations in root length and weight; while root development was inhibited in KO plants (Fig. 6E–G). This suggests that SlbHLH70 may be directly involved in the regulation of root development, enhancing the root system's ability to absorb water, thereby improving the drought resistance of plants.

From the perspective of conservative mechanisms, the regulation of the ABA signaling pathway by SlbHLH70 is consistent with the functions of bHLH factors in various plants, which is a conservative strategy for plants to cope with drought. In *A. thaliana*, AtbHLH112 activates the expression of genes related to ABA synthesis and signaling by binding to the G-box/E-box motifs of downstream genes, thereby enhancing the drought tolerance of plants. In rice, OsbHLH148 integrates JA and ABA signaling pathways to regulate drought response through interaction with OsJAZ proteins. This study found that SlbHLH70 can directly bind to the G-box motif (CACGTG) in the promoters of SlNCED1 (a key gene for ABA synthesis in tomato), SlPYL8 (an ABA receptor gene), and SlSnRK2.1 (a gene in the signaling pathway), upregulating their expression, while inhibiting the transcription of SlCYP707A2 (an ABA catabolic gene). Ultimately, this leads to a significant increase in endogenous ABA content in overexpressed lines (Fig. 5D and F). This regulatory pattern is completely consistent with the mechanism by which bHLH factors in the aforementioned species enhance drought tolerance through the ABA pathway, confirming that the ABA signaling pathway is the core conservative pathway through which transcription factors of the bHLH family regulate plant drought response. Moreover, the regulation of target gene expression by recognizing G-box motifs is the key molecular basis for realizing this conservative function.

**Figure 7:**
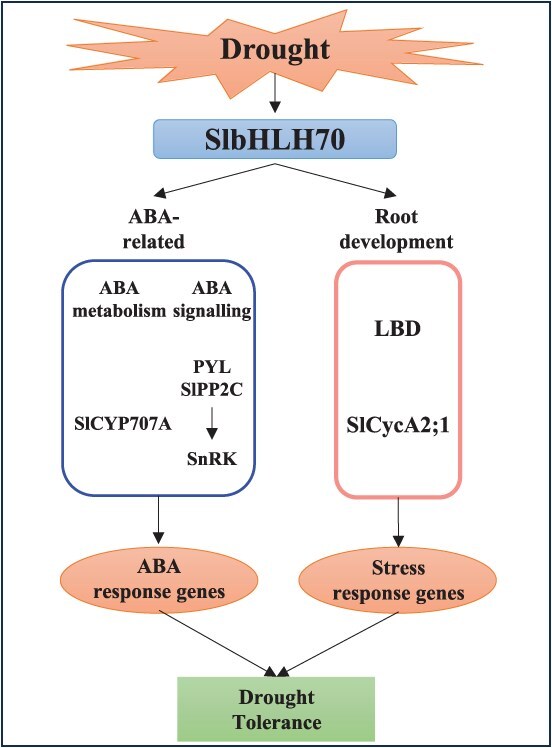
Proposal for the functional model of *SlbHLH70* in drought tolerance. Under drought stress, SlbHLH70 directly binds to the G-box motifs in the promoters of ABA biosynthesis-related genes (such as *SlNCED1*) and signal transduction-related genes (such as *SlPYL8*, *SlSnRK2.1*) to activate their transcription, thereby increasing the accumulation of endogenous ABA and downstream stress response cascades. Meanwhile, SlbHLH70 targets root development-related genes (such as *SlCycA2;1*, *SlLBD40*) to promote root growth and biomass accumulation, enhancing water absorption capacity. Conversely, knockout of SlbHLH70 disrupts ABA metabolic balance and root plasticity, leading to decreased drought tolerance.

This study systematically explored the role and mechanism of the tomato bHLH transcription factor gene SlbHLH70 in responding to drought stress. The results showed that SlbHLH70 has a significant impact on the drought resistance of tomato plants by regulating ABA synthesis, signal pathways, and root development (Fig. 7). The findings of this study not only provide a solid foundation for a deeper understanding of the molecular mechanisms underlying tomato responses to drought stress but also offer potential genetic resources for stress-resistant tomato breeding, thus holding important theoretical and practical significance.

## Materials and methods

### Materials and conditions for plant growth

The study utilized the tomato cultivar MT as both the subject and recipient of the transgene. Plants were cultivated in growth chambers with a 16-hour photoperiod at 25°C and an 8-hour dark period at 22°C, maintaining 80% relative humidity.

### Prediction of cis-acting elements in the gene promoters

The promoter sequences, located 3 kb upstream of the 5′ UTR, were extracted from the ITAG 2.4 genome annotation using the location and chromosome number of SlbHLH70 genes from the gff3 file. All promoter sequences were submitted to the PlantCARE database for cis-element prediction [51], and the outcomes were visualized using GSDS 2.0.

### Drought stress and hormone treatments for gene expression analysis

MT seeds that were surface-sterilized were sown on 1/2 MS medium plates for germination. Seedlings of uniform size were transferred from 1/2 MS medium plates to soil after 7 days of cultivation (each plant is planted individually, and the soil texture in each pot is uniform and of the same quality). Four-week-old tomato plants grown in pots with soil were selected for uniformity in growth status and plant size, followed by natural drought stress treatment at a constant room temperature of 25°C. Prior to stress initiation, all plants were fully irrigated in a water tank until soil reached field capacity, after which water supply was terminated. The control group received regular watering to maintain soil moisture at field capacity throughout the experiment. During the drought stress period, phenotypic alterations of the plants were visually monitored and documented daily. To assess the expression pattern of SlbHLH70 under exogenous hormone treatments, 4-week-old seedlings grown in soil were subjected to foliar application of 10 mM PEG, 10 μM ABA, or 10 μM MeJA. The plants were then incubated for 0, 2, 6, 12, and 24 hours prior to RNA extraction.

### Subcellular localization of SlbHLH70

The subcellular localization of SlbHLH70 was examined by cloning its full-length CDS, minus the stop codon, into the pCAMBIA1300-GFP vector under the control of the 35S promoter, creating the 35S-SlbHLH70-GFP construct. This construct and the empty pCAMBIA1300-GFP vector were used for transient transformation in *Nicotiana benthamiana* via Agrobacterium infiltration. A red fluorescent protein (RFP) fused with the nuclear marker HY5 was co-expressed to allow for nuclear imaging. After 48 hours, the fluorescence signals from the GFP protein expressed in epidermal cells were observed using a confocal laser scanning microscope.

### Tomato transformation

Total RNA was extracted from tomato and used for first-strand cDNA synthesis, followed by PCR amplification of the full-length CDS of SlbHLH70 using gene-specific primers. The amplified fragment was inserted into the plant expression vector pFGC5941. The constructed vector was then transformed into the tomato variety MT via Agrobacterium-mediated transformation (Agrobacterium strain GV3101) with tissue culture support.

### Extraction of RNA and subsequent qRT-PCR analysis were conducted

RNA extraction from MT tomato leaves was performed using TRIzol reagent (Tiangen, China). Subsequently, first-strand cDNA was synthesized using the M-MLV Reverse Transcriptase kit (Vazyme, China). Quantitative real-time PCR (qRT-PCR) was conducted using SYBR qPCR Master Mix (Vazyme, China), and gene expression levels were determined by the 2^−ΔΔCt^ method. The SlACTIN1 gene was selected as the reference gene for normalization. Supplementary Table S4 contains the primer sequences utilized in this study.

### Measurement of internal ABA and JA levels

Leaves from 35-day-old MT plants were collected for the measurement of endogenous ABA and JA content. Tomato leaves from OE, KO, and WT lines were flash-frozen in liquid nitrogen and stored at −80°C for subsequent processing. A total of three independent biological replicates were collected for each line. Endogenous ABA and JA levels were determined by Conwinced-Test Technology Co., Ltd., Nanjing, China [52].

### DAP-seq assay

The DAP-seq binding assay was performed following established protocols [27, 53]. Genomic DNA was extracted from young leaves of MT tomatoes and fragmented to an average size of 200 bp using a Covaris M220 sonicator. After centrifugation, the DNA-containing supernatant was taken, and sequencing libraries were prepared using the MICH TLX DNA Sequencing Kit (catalog #NGS0602, Lanjing Biotechnology). Concurrently, the coding sequence of SlbHLH70 was cloned into the pFN19K HaloTag T7 SP6 Flexi vector, and the recombinant protein was purified with Magne HaloTag Beads (Promega). For the DNA–protein binding reaction, 50 ng of adapter-ligated genomic DNA fragments and protein-bound beads were incubated in 50 μl of wash/binding buffer with rotation at room temperature for 60 minutes. After removing unbound DNA by washing three times with the same buffer, the bound DNA was eluted by resuspending the beads in 30 μl of elution buffer and heating at 98°C for 10 minutes. After centrifugation, 25 μl of the supernatant was used for PCR amplification with KAPA HiFi HotStart ReadyMix (Roche) for 10 cycles. The PCR products were purified, size-selected using MICH DNA Clean Beads (catalog #NGS0201, Lanjing Biotechnology), and dissolved in 20 μl of nuclease-free water. Sequencing was performed on the Illumina NovaSeq platform. The negative control libraries were prepared in the same way but without adding the recombinant protein.

### EMSA

The coding sequence of SlbHLH70 was cloned into an expression vector, and the recombinant vector was transformed into *Escherichia coli* Rosetta [54]. After culturing to OD600 ≈ 0.6, protein expression was induced with 0.5 mM IPTG at 16°C/37°C for 16 hours. The recombinant His-bHLH70 protein was purified from bacterial lysate supernatant. Biotin-labeled probes (WT, mutant, competitor) were synthesized; probes >60 bp were obtained via PCR amplification and gel recovery, while 11- to 59-bp probes were formed by annealing. EMSA was performed in a 20-μl system containing purified protein, labeled probe, and binding buffer (Beyotime, GS005), incubated at 25°C for 30 minutes (20×/50× unlabeled probe was added for competition assays). Samples were resolved by 6% non-denaturing PAGE at 80 V on ice, transferred to a nylon membrane, cross-linked, blocked, and incubated with HRP-conjugated streptavidin (Proteintech, SA00001-0). Signals were detected with ECL developing solution (Advansta, K-12045-D50) using a chemiluminescent imaging system (Tanon, 5200). Probe sequences are listed in Supplementary Table S4.

### Statistical analysis

Results are presented as mean values with standard deviations. Statistical significance was determined by one-way ANOVA (Tukey's test) using GraphPad Prism (version 8.0.1, USA). A *P*-value of less than 0.05 was deemed statistically significant. Significant differences in mean values, as determined by Student's *t* test, are marked with asterisks: *P < 0.05 and **P < 0.01.

## Supplementary Material

Web_Material_uhag075

## Data Availability

The RNA-seq data supporting the findings of this study have been deposited in the CNGB Nucleotide Sequence Archive (CNSA) (https://www.cngb.org/), under project number CNP0009185.

## References

[1] Zhang H, Zhu J, Gong Z. et al. Abiotic stress responses in plants. Nat Rev Genet. 2022;23:104–19 34561623 10.1038/s41576-021-00413-0

[2] Mccue KF, Hanson AD. Drought and salt tolerance: towards understanding and application. Trends Biotechnol. 1990;8:358–62

[3] Liu Y, Xin X, Zheng J. et al. SlSAMS1 improves carbon and nitrogen metabolism in tomato under salt stress. Vegetable Research. 2025;5:e021

[4] Zhao H, Shin D, Zhu Y. et al. Bridging the knowledge gap: utilization of mediator subunits for crop improvement. Plant Cell Environ. 2025;48:213–25 39254322 10.1111/pce.15142

[5] Zhang J, Jia W, Yang J. et al. Role of ABA in integrating plant responses to drought and salt stresses. Field Crop Res. 2006;97:111–9

[6] Feller A, Machemer K, Braun EL. et al. Evolutionary and comparative analysis of MYB and bHLH plant transcription factors. Plant J. 2011;66:94–116 21443626 10.1111/j.1365-313X.2010.04459.x

[7] Carretero-Paulet L, Galstyan A, Roig-Villanova I. et al. Genome-wide classification and evolutionary analysis of the bHLH family of transcription factors in Arabidopsis, poplar, rice, moss, and algae. Plant Physiol. 2010;153:1398–412 20472752 10.1104/pp.110.153593 PMC2899937

[8] Blanc-Mathieu R, Dumas R, Turchi L. et al. Plant-TFClass: a structural classification for plant transcription factors. Trends Plant Sci. 2024;29:40–51 37482504 10.1016/j.tplants.2023.06.023

[9] Pires N, Dolan L. Origin and diversification of basic-helix–loop–helix proteins in plants. Mol Biol Evol. 2010;27:862–74 19942615 10.1093/molbev/msp288 PMC2839125

[10] Gao F, Dubos C. The Arabidopsis bHLH transcription factor family. Trends Plant Sci. 2023;29:668–80 38143207 10.1016/j.tplants.2023.11.022

[11] Ding W, Yu Z, Tong Y. et al. A transcription factor with a bHLH domain regulates root hair development in rice. Cell Res. 2009;19:1309–11 19752888 10.1038/cr.2009.109

[12] Hatakeyama J, Kageyama R. Retinal Cell Fate Determination and bHLH Factors. In:Seminars in Cell & Developmental Biology. 2004, pp. 83–9 10.1016/j.semcdb.2003.09.005 15036211

[13] Guo J, Sun B, He H. et al. Current understanding of bHLH transcription factors in plant abiotic stress tolerance. Int J Mol Sci. 2021;22:4921 34066424 10.3390/ijms22094921 PMC8125693

[14] Lei P, Jiang Y, Zhao Y. et al. Functions of basic helix–loop–helix (bHLH) proteins in the regulation of plant responses to cold, drought, salt, and iron deficiency: a comprehensive review. J Agric Food Chem. 2024;72:10692–709 38712500 10.1021/acs.jafc.3c09665

[15] Liang B, Wan S, Ma Q. et al. A novel bHLH transcription factor PtrbHLH66 from trifoliate orange positively regulates plant drought tolerance by mediating root growth and ROS scavenging. Int J Mol Sci. 2022;23:15053 36499381 10.3390/ijms232315053 PMC9740576

[16] Song Y, Li S, Sui Y. et al. SbbHLH85, a bHLH member, modulates resilience to salt stress by regulating root hair growth in sorghum. Theor Appl Genet. 2022;135:201–16 34633473 10.1007/s00122-021-03960-6

[17] Iuchi S, Kobayashi M, Taji T. et al. Regulation of drought tolerance by gene manipulation of 9-cis-epoxycarotenoid dioxygenase, a key enzyme in abscisic acid biosynthesis in Arabidopsis. Plant J. 2001;27:325–33 11532178 10.1046/j.1365-313x.2001.01096.x

[18] Schwartz SH, Qin X, Zeevaart JA. Elucidation of the indirect pathway of abscisic acid biosynthesis by mutants, genes, and enzymes. Plant Physiol. 2003;131:1591–601 12692318 10.1104/pp.102.017921 PMC1540303

[19] Matilla AJ, Carrillo-Barral N, Rodríguez-Gacio MDC. An update on the role of NCED and CYP707a ABA metabolism genes in seed dormancy induction and the response to after-ripening and nitrate. J Plant Growth Regul. 2015;34:274–93

[20] Quinet M, Angosto T, Yuste-Lisbona FJ. et al. Tomato fruit development and metabolism. Front Plant Sci. 2019;10:1554 31850035 10.3389/fpls.2019.01554 PMC6895250

[21] Zhu Y, Zhu G, Xu R. et al. A natural promoter variation of SlBBX31 confers enhanced cold tolerance during tomato domestication. Plant Biotechnol J. 2023;21:1033–43 36704926 10.1111/pbi.14016 PMC10106858

[22] Wang Y, Wang H, Xin Y. et al. Genome-wide identification of SmJAZ gene family in eggplant and functional mechanism analysis of SmJAZ9 modulating high temperature and darkness stress-regulated anthocyanin biosynthesis. Vegetable Research. 2025;5

[23] Lin T, Zhu G, Zhang J. et al. Genomic analyses provide insights into the history of tomato breeding. Nat Genet. 2014;46:1220–6 25305757 10.1038/ng.3117

[24] Heim MA, Jakoby M, Werber M. et al. The basic helix–loop–helix transcription factor family in plants: a genome-wide study of protein structure and functional diversity. Mol Biol Evol. 2003;20:735–47 12679534 10.1093/molbev/msg088

[25] Zhang J, Huang Q, Zhong S. et al. Sperm cells are passive cargo of the pollen tube in plant fertilization. Nat Plants. 2017;3:1–5 10.1038/nplants.2017.79 PMC5960590 28585562

[26] Karas B, Amyot L, Johansen C. et al. Conservation of lotus and Arabidopsis basic helix–loop–helix proteins reveals new players in root hair development. Plant Physiol. 2009;151:1175–85 19675148 10.1104/pp.109.143867 PMC2773103

[27] Bartlett A, O'Malley RC, Huang SC. et al. Mapping genome-wide transcription-factor binding sites using DAP-seq. Nat Protoc. 2017;12:1659–72 28726847 10.1038/nprot.2017.055 PMC5576341

[28] Ramírez F, Ryan DP, Grüning B. et al. DeepTools2: a next generation web server for deep-sequencing data analysis. Nucleic Acids Res. 2016;44:W160 27079975 10.1093/nar/gkw257 PMC4987876

[29] Sun Y, Han Y, Sheng K. et al. Single-cell transcriptomic analysis reveals the developmental trajectory and transcriptional regulatory networks of pigment glands in *Gossypium bickii*. Mol Plant. 2023;16:694–708 36772793 10.1016/j.molp.2023.02.005

[30] Zhao H, Yang M, Bishop J. et al. Identification and functional validation of super-enhancers in *Arabidopsis thaliana*. Proc Natl Acad Sci. 2022;119:e2079639177 10.1073/pnas.2215328119 PMC9860255 36409894

[31] Zhao Y, Chan Z, Gao J. et al. ABA receptor PYL9 promotes drought resistance and leaf senescence. Proc Natl Acad Sci. 2016;113:1949–54 26831097 10.1073/pnas.1522840113 PMC4763734

[32] Liu L, Zhang J, Xu J. et al. SlMYC2 promotes SlLBD40-mediated cell expansion in tomato fruit development. Plant J. 2024;118:1872–88 38481350 10.1111/tpj.16715

[33] Shahid A, White G, Diuwe J. et al. SLMAD: Statistical learning-based metric anomaly detection. In: International Conference on Service-Oriented Computing. Cham: Springer International Publishing, 2020, fanyu

[34] Seo JS, Joo J, Kim MJ. et al. OsbHLH148, a basic helix–loop–helix protein, interacts with OsJAZ proteins in a jasmonate signaling pathway leading to drought tolerance in rice. Plant J. 2011;65:907–21 21332845 10.1111/j.1365-313X.2010.04477.x

[35] Kovak E, Blaustein-Rejto D, Qaim M. Genetically modified crops support climate change mitigation. Trends Plant Sci. 2022;27:627–9 35148945 10.1016/j.tplants.2022.01.004

[36] Li Z, Liu C, Zhang Y. et al. The bHLH family member ZmPTF1 regulates drought tolerance in maize by promoting root development and abscisic acid synthesis. J Exp Bot. 2019;70:5471–86 31267122 10.1093/jxb/erz307 PMC6793450

[37] Liu W, Tai H, Li S. et al. B HLH 122 is important for drought and osmotic stress resistance in a rabidopsis and in the repression of ABA catabolism. New Phytol. 2014;201:1192–204 24261563 10.1111/nph.12607

[38] Liang Y, Ma F, Li B. et al. A bHLH transcription factor, SlbHLH96, promotes drought tolerance in tomato. Hortic Res. 2022;9:uhac198 36467272 10.1093/hr/uhac198 PMC9714257

[39] Waseem M, Li Z. Overexpression of tomato SlbHLH22 transcription factor gene enhances fruit sensitivity to exogenous phytohormones and shortens fruit shelf-life. J Biotechnol. 2019;299:50–6 31054298 10.1016/j.jbiotec.2019.04.012

[40] Hao Y, Zong X, Ren P. et al. Basic helix–loop–helix (bHLH) transcription factors regulate a wide range of functions in Arabidopsis. Int J Mol Sci. 2021;22:7152 34281206 10.3390/ijms22137152 PMC8267941

[41] Li J, Wang T, Han J. et al. Genome-wide identification and characterization of cucumber bHLH family genes and the functional characterization of CsbHLH041 in NaCl and ABA tolerance in Arabidopsis and cucumber. BMC Plant Biol. 2020;20:1–20 32527214 10.1186/s12870-020-02440-1 PMC7291561

[42] Pireyre M, Burow M. Regulation of MYB and bHLH transcription factors: a glance at the protein level. Mol Plant. 2015;8:378–88 25667003 10.1016/j.molp.2014.11.022

[43] Liu Y, Ji X, Nie X. et al. Arabidopsis Atb HLH 112 regulates the expression of genes involved in abiotic stress tolerance by binding to their E-box and GCG-box motifs. New Phytol. 2015;207:692–709 25827016 10.1111/nph.13387

[44] Lian T, Xu Y, Li L. et al. Crystal structure of tetrameric Arabidopsis MYC2 reveals the mechanism of enhanced interaction with DNA. Cell Rep. 2017;19:1334–42 28514654 10.1016/j.celrep.2017.04.057

[45] Zhu C, Lin Z, Liu Y. et al. A bamboo bHLH transcription factor PeRHL4 has dual functions in enhancing drought and phosphorus starvation tolerance. Plant Cell Environ. 2024;47:3015–29 38644587 10.1111/pce.14920

[46] Hu C, Wang M, Zhu C. et al. A transcriptional regulation of ERF15 contributes to ABA-mediated cold tolerance in tomato. Plant Cell Environ. 2024;47:1334–47 38221812 10.1111/pce.14816

[47] Chong L, Xu R, Huang P. et al. The tomato OST1–VOZ1 module regulates drought-mediated flowering. Plant Cell. 2022;34:2001–18 35099557 10.1093/plcell/koac026 PMC9048945

[48] de Ollas C, Dodd IC. Physiological impacts of ABA–JA interactions under water-limitation. Plant Mol Biol. 2016;91:641–50 27299601 10.1007/s11103-016-0503-6 PMC4932129

[49] Miyamoto K, Shimizu T, Mochizuki S. et al. Stress-induced expression of the transcription factor RERJ1 is tightly regulated in response to jasmonic acid accumulation in rice. Protoplasma. 2013;250:241–9 22456953 10.1007/s00709-012-0400-z

[50] Fu J, Wu H, Ma S. et al. OsJAZ1 attenuates drought resistance by regulating JA and ABA signaling in rice. Front Plant Sci. 2017;8:2108 29312378 10.3389/fpls.2017.02108 PMC5733117

[51] Rombauts S, Déhais P, Van Montagu M. et al. PlantCARE, a plant cis-acting regulatory element database. Nucleic Acids Res. 1999;27:295–6 9847207 10.1093/nar/27.1.295 PMC148162

[52] Pan X, Welti R, Wang X. Quantitative analysis of major plant hormones in crude plant extracts by high-performance liquid chromatography–mass spectrometry. Nat Protoc. 2010;5:986–92 20448544 10.1038/nprot.2010.37

[53] O’Malley RC, Huang SC, Song L. et al. Cistrome and epicistrome features shape the regulatory DNA landscape. Cell. 2016;165:1280–92 27203113 10.1016/j.cell.2016.04.038 PMC4907330

[54] Li Q, Zhou L, Chen Y. et al. Phytochrome interacting factor regulates stomatal aperture by coordinating red light and abscisic acid. Plant Cell. 2022;34:4293–312 35929789 10.1093/plcell/koac244 PMC9614506

