# Supplementary material for: Transcription factor SlbHLH70 enhances drought tolerance in tomato
Source: Hortic Res. 2026 Mar 5;13(6):uhag075. doi: 10.1093/hr/uhag075 (PMC13253334; doi:10.1093/hr/uhag075)
Supplement: Web_Material_uhag075 [file web_material_uhag075.zip › 03 Supplementary Information.docx]

**Supplementary Tables**

**Table S1** Cis-elements information of *SlbHLH70* in promoter region.

**Table S2** A list of candidate motifs of *SlbHLH70* identified by MEME-ChIP.

**Table S3** Differentially expressed genes of WT and *SlbHLH70* knockout plants before and after drought stress.

**Table S4** Primers used in this study.

**Supplementary Figures**


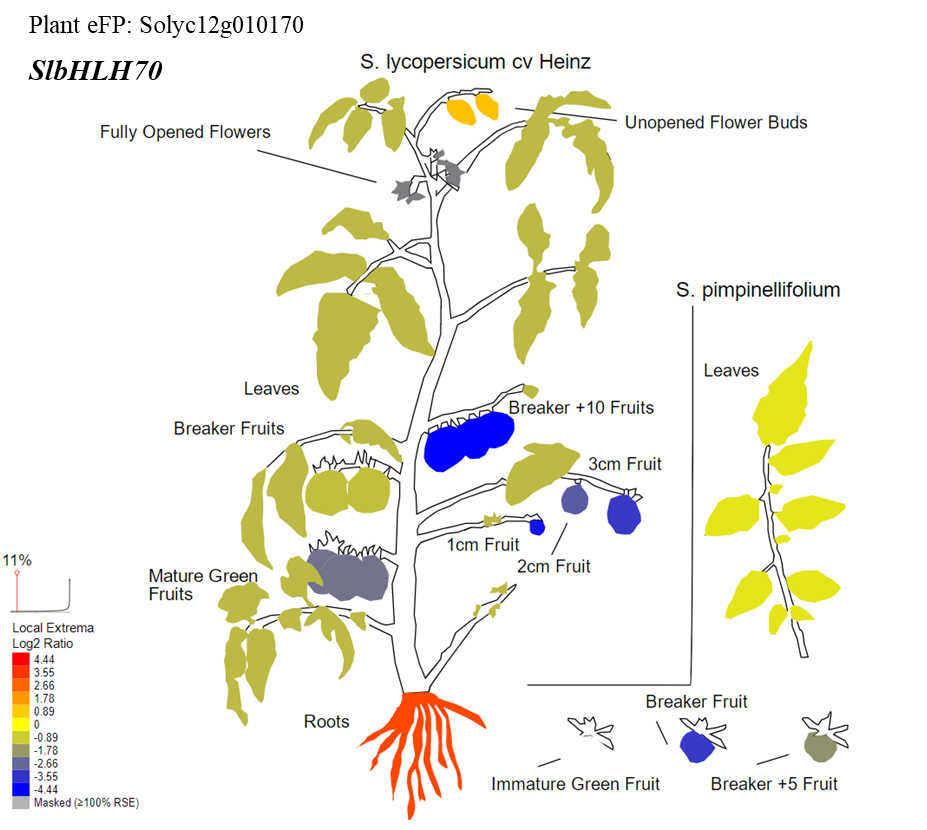


**Fig. S1** Tissue specific expression patterns of SlbHLH70. Data are Illumina-derived and RPKM-normalized. These images were generated with the Plant eFP at bar.utoronto.ca/eplant as described by by Waese et al. (2017).


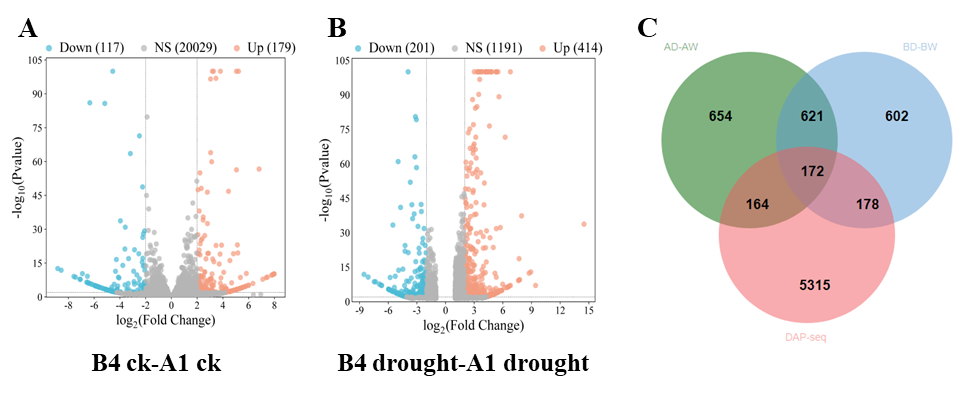


**Fig. S2** Identification of drought-responsive genes directly regulated by *SlbHLH70.* (A-B) Volcano plot of differentially expressed genes (DEG) in SlbHLH70 knockout line (KO4) and wild-type (WT) plants under normal water (A) and drought stress (B) conditions (| log FC | ≥ 2, padj ≤ 0.01). The red/blue dots represent up/down-regulated genes. (C) Comprehensive analysis of DAP-seq peaks and RNA-seq data. 164 genes with a SlbHLH70 binding promoter and drought response expression that differ before and after knockout of the SlbHLH70 gene are highlighted. Venn diagram. "AD-AW" indicates DEGs between drought-stressed and normally watered plants; "BD-BW" indicates DEGs between drought-stressed and normally watered plants; "DAP-seq", a potential target gene from DAP-seq.


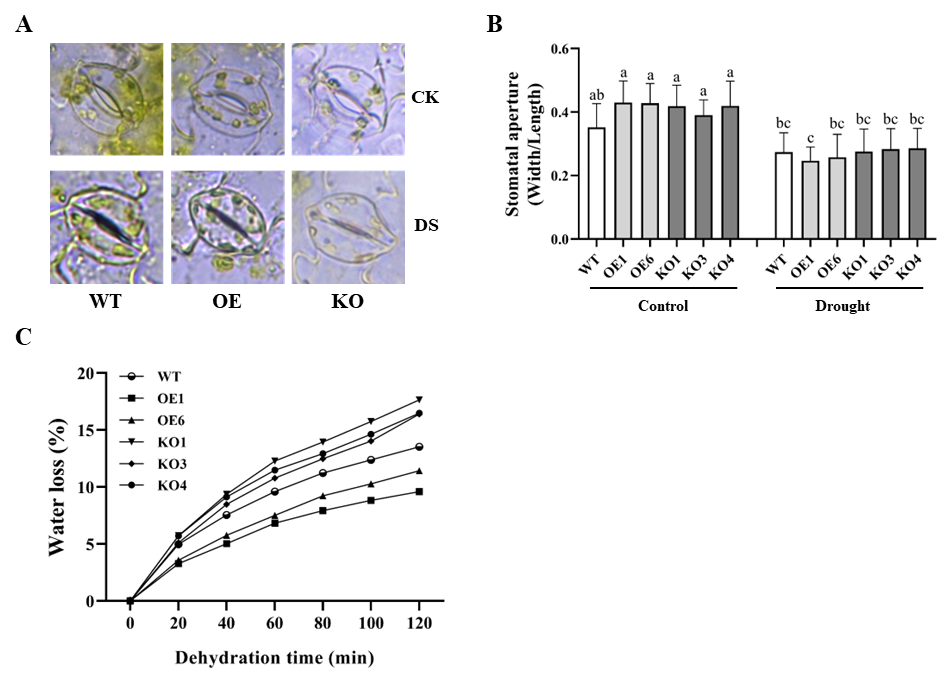


**Fig. S3** (A,B) Stomatal aperture measurements in WT and transgenic plants. No significant differences observed (P > 0.05). (C) Water loss rates of detached leaves from WT, OE, and KO plants.
